# Facts and Fancies

**Published:** 1897-03

**Authors:** 


### FACTS AND FANCIES.

The house-cat has added another burden of responsibility, having been the agent of transmission of smallpox from one house to another.

The Pasteur Institute of Paris reports one case of rabies in every twenty treated the result of cat-bites.

Dr. Webb's famous hackney stallion "Matchless," of Londesboro, brought $12,000, and was purchased by Mr. Walter L. Clark, a noted hackney breeder.

Baroness Hirsch has recently given to the Pasteur Institute of France the munificent sum of $50,000 for the furthering of the important work of this institution.

The annual meeting of the Iowa State Veterinary Medical Association, in January, was a very successful gathering.

The Minnesota Legislature has been asked for an appropriation of $18,000 for the erection on their State fair-grounds of an amphitheatre seating 10,000 people, to judge their live-stock in.

WANTED.—Position as assistant by a graduate who had some experience. Address Doctor, care of A. Eger, 57 East Jackson St., Chicago, Illinois.

